# Effect of alkaline mineral complex buffer addition on milk performance, blood parameters, and rumen microbiota in early-lactation dairy cows

**DOI:** 10.1128/spectrum.00239-25

**Published:** 2025-11-05

**Authors:** Xiaoting Zhou, Cheng Guo, Xiaowei Wang, Fanlin Kong, Shengli Li

**Affiliations:** 1 State Key Laboratory of Animal Nutrition and Feeding, Beijing Engineering Technology Research Center of Raw Milk Quality and Safety Control, College of Animal Science and Technology, China Agricultural University 34752, Beijing, China; 2 Feed Research Institute, Chinese Academy of Agricultural Sciences 12661, Beijing, China; 3 College of Animal Science and Technology, Ningxia University 56693, Yinchuan, China; Luonnonvarakeskus, Oulu, Finland

**Keywords:** early-lactation dairy cows, alkaline mineral complex buffer, rumen microbiota

## Abstract

This study evaluated the effects of an alkaline mineral complex buffer (AMCB) in the diet on lactation performance, blood parameters, rumen fermentation, and microbial composition in early-lactation Holstein cows. In total, 30 Holstein cows (average parity 3.76 ± 0.48, milk in lactation day 22.45 ± 0.51) were randomly assigned to a control group (CON) or an experimental group (EXP) receiving 50 mL AMCB daily, providing 10 g sodium metasilicate, 5 g potassium bicarbonate, 0.5 mg zinc oxide, and 0.05 mg Ge-132 per cow. Cows supplemented with AMCB exhibited higher dry matter intake, milk production, fat-corrected milk yield, and energy-corrected milk yield, as well as increased lactose and milk protein concentrations and daily yields (*P* < 0.05) compared to the CON group. Notably, there was a significant reduction in somatic cell counts in the milk (*P* < 0.05). AMCB supplementation also led to a decrease in serum levels of aspartate aminotransferase and alanine aminotransferase (*P* < 0.05), while increasing rumen ammonia nitrogen concentrations (*P* < 0.05). A trend toward greater bacterial richness (Chao1, *P* = 0.06) was observed, with significant increases in *Prevotellaceae*_UCG-001 and *Ruminococcaceae*_NK4A214_group by d 30 (*P* < 0.05), and *Enterococcus* identified as a biomarker in the EXP group. These findings underscore that AMCB supplementation enhances production performance in dairy cows, mitigates liver function impairment, and stimulates the proliferation of protein-degrading bacteria in the rumen. This highlights AMCB’s potential as a strategic dietary intervention, offering valuable guidance for optimizing nutritional management to support both metabolic health and productivity for early-lactation cows.

**IMPORTANCE:**

Early lactation is a critical period for dairy cows, characterized by high milk production demands and a heightened risk of subacute ruminal acidosis, which compromises rumen function, fiber digestion, and overall productivity. While research on buffering agents has predominantly focused on monogastric animals or single-agent applications, the role of composite buffers in early-lactation dairy cows remains underexplored. Our study demonstrated that supplementation with an alkaline mineral complex buffer (AMCB) significantly improved milk yield and quality, increased the relative abundance of protein-degrading bacteria, reduced somatic cell counts, and enhanced liver function. These results provide compelling evidence supporting the strategic application of composite buffering agents to enhance lactational performance and metabolic health in dairy cows.

## INTRODUCTION

The global population is projected to reach 9.8 billion by 2050 (1), driving increased demand for food and raising expectations for the efficiency of primary production systems. Over the past three decades, milk production has surged by nearly 64% (2), largely due to genetic advancements in dairy cows and diets enriched with highly fermentable carbohydrates, which have enhanced productivity (3). However, balancing diets to maximize milk yield while maintaining adequate effective fiber for rumen stability remains a challenge. Physically effective neutral detergent fiber (peNDF) plays a vital role in rumen motility, chewing, rumination, and saliva production, acting as a natural buffer (4). Nevertheless, fiber alone is insufficient to meet the high energy demands of high-producing cows (5). To compensate, producers increase concentrate levels, incorporating rapidly fermentable carbohydrates such as corn, wheat, and sorghum. These carbohydrates quickly convert into volatile fatty acids (VFAs), especially propionate, the primary energy source for milk production (6). However, excessive VFA accumulation can overwhelm the rumen’s buffering capacity, leading to a rapid pH drop (7), which disrupts the microbial populations responsible for fiber degradation (8). When rumen pH falls below 6.3, digestibility of acid detergent fiber (ADF) decreases by 3.6% (9), compromising both fiber digestion and milk composition, with potential health consequences like diarrhea, inflammation, and liver abscesses (10, 11). During the early lactation period, dairy cows undergo a rapid dietary transition from high-forage to high-concentrate diets to meet elevated energy demands for milk production. This abrupt shift often exceeds the adaptive capacity of the rumen, leading to incomplete remodeling of the ruminal epithelium (12). The resulting impaired ability to handle elevated VFA production increases the risk of subacute ruminal acidosis (SARA) and predisposes cows to metabolic disorders such as ketosis (13). Notably, multiparous cows face a 30% incidence of SARA during this critical phase (14). This imbalance between dietary fermentability and ruminal adaptation compromises rumen health and overall metabolic stability.

It is widely recognized that incorporating rumen buffers, such as sodium bicarbonate and other alkali salts, into the diets of early-lactation dairy cows has become a globally adopted practice to mitigate metabolic challenges (15), effectively preventing low rumen pH and minimizing production losses. While recent studies have highlighted alternative agents, such as zeolite (16, 17), clay (18), alkaline metal oxides (19, 20), and lithothamnion (calcified algae) (15), which can also stabilize the rumen environment with more sustained buffering effects. Zeolite and clay, both belonging to silicates, can capture cations like NH_4_^+^, slowing their absorption and release, which improves microbial nitrogen utilization and reduces nitrogen excretion in manure (21–23). Zinc oxide (ZnO) (more accurately described as a rumen alkalizer) dissolves, neutralizes excess H^+^, raises rumen pH, and prevents dysfunction. In monogastric animals, zinc supplementation has been shown to enhance gut integrity, support microbial balance, and improve growth performance (24–26). Notably, composite buffers incorporating mineral cations like sodium and potassium have shown potential to influence ruminal biohydrogenation and milk composition beyond their buffering capacity (27–29). Studies also suggest that combining buffers, such as a 3:1 ratio of sodium bicarbonate to magnesium oxide, is more effective in promoting ruminal health and performance than single agents (30). Despite these advancements, the combined efficacy of emerging buffering agents and their interactions with rumen function and microbial composition during peak lactation remains insufficiently studied.

The alkaline mineral complex buffer (AMCB) examined in this study—comprising sodium metasilicate (5H₂O·Na₂SiO₃), potassium bicarbonate (KHCO₃), ZnO, and Ge-132—offers a novel composite formulation that has shown promise in previous studies (31, 32). AMCB has been demonstrated to improve gut health, antioxidant defenses, and immune function in weaned piglets (33, 34) and neonatal calves (35), as well as to enhance postpartum dry matter intake (DMI), production performance, and rumen microbiota in periparturient dairy cows (36). While these benefits are well documented in monogastric animals and specific physiological stages of ruminants, there is a notable research gap regarding their application in peak-lactation dairy cows. Given the critical metabolic challenges and rumen health demands during this period, further investigation into the potential benefits of the alkaline composite buffer in improving performance and health during early lactation is essential. Therefore, our study evaluated the effects of AMCB supplementation on milk production, blood metabolism, rumen fermentation, and bacterial diversity in dairy cows during early lactation. We hypothesize that adding AMCB in the diet may enhance lactational performance and support overall health in early-lactation dairy cows. These findings provide evidence-based recommendations for optimizing feeding strategies to support dairy cows during the critical early-lactation phase, ultimately contributing to more efficient and sustainable dairy farming practices.

## MATERIALS AND METHODS

### Experimental design and diets

The experiment was carried out in 2022 at a large-scale commercial dairy farm in Yinchuan, Ningxia, China. In all, 30 Holstein cows, with an average parity of 3.76 ± 0.48 and 22.45 ± 0.51 d in milk lactation, were selected as individual experimental units. The cows were randomly allocated into two groups using a completely randomized design: the treatment group (EXP), supplemented with AMCB, and the control group (CON), without AMCB supplementation.

The diet for both groups was formulated based on the Nutrient Requirements of Dairy (37), with composition and nutrient contents displayed in Table 1. Each cow in the supplemented group received 50 mL of AMCB at 0730 h daily, sourced from Nail Biotechnology Company, Beijing, China, and the detailed formula of AMCB is provided in Table 2. Since AMCB is a water-soluble colloidal substance, it was diluted in a 1:4 ratio with water, following previous studies (38). Prior to the distribution of the basal total mixed ration (TMR), the diluted AMCB was mixed with a small amount of fresh TMR and offered directly to each cow for individual consumption. The experiment was conducted on a tie-stall barn dairy farm, consisting of an 8-d adaptation period followed by a 30-d experimental phase. Cows were fed twice daily at 07:30 and 15:30 h, with milking performed three times a day at 06:30, 14:30, and 20:30 h. Drinking water, supplied exclusively as tap water to minimize mineral interference, was provided *ad libitum* throughout the study.

**TABLE 1:** Composition and nutrient levels of the experimental diets

Ingredient, % of diet DM	Content	Nutrient compositions^*c*^ (% of dry matter unless noted)		Content
Corn silage	32.37	Dry matter (% of fresh weight),%		48.57 ± 2.21
Alfalfa hay	11.73	NE_L_, Mcal/kg		1.63
Cotton seed	4.73	Crude protein, %		16.29 ± 0.12
Steam-flaked corn	2.69	Neutral detergent fiber, %		30.16 ± 1.75
Corn	17.53	Acid detergent fiber, %		23.05 ± 1.25
Wheat bran	3.92	Lignin, %		3.73 ± 0.36
Soybean meal	9.71	Starch, %		26.21 ± 0.34
Cottonseed meal	2.97	Ether extract, %		5.87 ± 0.23
Corn distillers dried grains with solubles	9.04	Ash, %		9.45 ± 0.32
Expanded soybean	1.10			
Calcium bicarbonate	0.95			
Sodium bicarbonate	0.72			
Commercial premix^*a*^	0.19			
Commercial premix^*b*^	2.36			

^*a*^ Commercial premix1 (Hua Sheng Mu Ge Feed Co., Ltd., Yinchuan, China).

^*b*^ Commercial premix2 (Mengtai Dadi Biotechnology Development Co., Ltd., Hohhot, China).

^*c*^ NE_L_ was calculated based on National Research Council (2001) guidelines, and the other indicators were measured values.

**TABLE 2:** The composition of alkaline mineral complex buffer concentrate (34)

Ingredient	Content	Chemical formula
Sodium metasilicate pentahydrate	200 g/L	5H_2_O·Na_2_SiO_3_
Potassium bicarbonate	100 g/L	KHCO_3_
Zinc oxide	10 mg/L	ZnO
Bis-(carboxyethyl germanium) sesquioxide	1 mg/L	Ge-132

### Intake, milk performance

Dry matter intake (DMI) was calculated as the difference between the dry weight of feed offered and refusals for each cow. To avoid feed competition, cows were housed in individual stalls separated by railings. Feed offered and refusals were recorded daily, and DMI was calculated on a daily basis and summarized weekly. To determine the dry matter content of the diet, weekly TMR samples were collected, oven-dried at 65°C for 24 hours, and subsequently air-dried prior to nutritional analysis (39). Neutral detergent fiber (NDF) and ADF contents were determined using Van Soest’s method (40), while ether extract (EE) and crude protein (CP) were analyzed following the procedures outlined by the Association of Official Agricultural Chemists (AOAC) (41). Starch content was measured by a colorimetric method (36).

Milk yield was recorded daily for each cow and summarized weekly. Fat-corrected milk (4% FCM) and energy-corrected milk (ECM) were calculated according to the equations provided by Liu and Vandehaar (42). Milk was sampled from each cow at 06:30, 14:30, and 20:30 h on d 1, 15, and 30 of the experiment, respectively. The samples were mixed in a 4:4:3 ratio and preserved with potassium dichromate for subsequent analysis. Routine milk parameters, including lactose, fat, protein content, and somatic cell count (SCC), were measured with a milk composition analyzer. With commercial test kits (Nanjing Jiancheng Co., Ltd., Nanjing, China), the concentrations of milk urea nitrogen (MUN) were determined.

### Blood metabolites, liver enzymes

On d 1, 15, and 30 of the experimental period, blood samples were collected from 10 randomly assigned cows in each group. Using a non-anticoagulant vacuum tube, 10 mL of blood was drawn from the coccygeal vein 2 h post-morning feeding. The samples were allowed to coagulate for 30 min before being centrifuged for 15 min at 4°C at 3,000 × *g*. For further examination, the supernatant was collected and stored at −20°C. Bovine-specific enzyme-linked immunosorbent assay (ELISA) kits (Abcam, Cambridge, UK) and a microplate reader (Thermo Multiskan Ascent, Thermo Fisher Scientific, Shanghai, China) were employed to determine the serum concentrations of non-esterified fatty acids (NEFA), β-hydroxybutyrate (BHBA), immunoglobulin G and M (IgG, IgM), granulocyte-macrophage colony-stimulating factor (GM-CSF), and interleukin-6 (IL-6). Insulin levels were quantified using a multi-tube radioimmunoassay counter (BFM-96, Zhongcheng Electromechanical Technology Co., China). Additional biochemical indices related to nitrogen and energy metabolism, such as total triglyceride (TG), total cholesterol (TC), blood urea nitrogen (BUN), total protein (TP), albumin (ALB), and creatinine (Cr), were quantified using commercial kits (Nanjing Jiancheng Co., Ltd., Nanjing, China) and an automatic biochemical analyzer (CLS880, Zecen Biotechnology Co., Ltd., Taizhou, China). Indicators of oxidative stress, including superoxide dismutase (SOD), catalase (CAT), hydrogen peroxide (H_2_O_2_), and malondialdehyde (MDA), along with liver function markers such as total bilirubin (T-BIL), aspartate aminotransferase (AST), alanine aminotransferase (ALT), and alkaline phosphatase (ALP), were also measured using the same instrumentation.

### Rumen fermentation parameters

On d 1, 15, and 30 of the experimental period, eight cows from each group were chosen at random for rumen fluid collection. Rumen fluid was collected using a stomach tube connected to a vacuum pump 2 h before the midday feeding. To minimize salivary contamination, the initial 50 mL of rumen fluid was discarded. Approximately 1.8 mL of rumen fluid was immediately transferred to a 2 mL cryopreservation tube and preserved in a liquid nitrogen tank for subsequent rumen microbiota analysis. After undergoing four layers of gauze, a pH meter was used to determine the filtered rumen fluid’s pH, and 50 mL was retained for extra analysis at −20°C. The concentrations of VFAs were identified by gas chromatography-mass spectrometry (GC-MS) (TP-2060 System, Beijing Beifen Tianpu Instrument Technology Co., Ltd.), utilizing the procedure described by Zhang et al. (43). Rumen ammonia nitrogen (NH₃-N) concentration was determined by the colorimetric method of Broderick and Kang (44), and microbial crude protein (MCP) was quantified using the method of Makkar et al. (45) with a UV-1801 spectrophotometer (Beijing Rayleigh Analytical Instrument Co., Ltd., Beijing, China).

### DNA extraction, 16S rRNA gene sequencing, and bioinformatic analysis

DNA was extracted from rumen fluid samples using the Soil DNA Kit (M5635-02; OMEGA Bio-Tek, Norcross, GA, USA), and both the quantity and quality of the extracted DNA were evaluated using a Nanodrop spectrophotometer and 1.2% agarose gel electrophoresis, respectively. The V3-V4 hypervariable region of the bacterial 16S rRNA gene was amplified via PCR using forward primer 338F (5′ACTCCTACGGGGAGGCAGCA-3′) and reverse primer 806R (5′-GACTACHVGGGTWTCTAAT-3′), designed based on previous studies (46). The Illumina NovaSeq 6000 platform (Illumina, San Diego, CA, USA) was used to sequence the pure DNA, which was submitted to Shanghai Biozeron Technology Co., Ltd. (Shanghai, China).

Raw sequencing reads were subjected to quality filtering using FASTP (v0.20.0 [47]) to remove low-quality reads and adapter sequences. High-quality sequences were then merged using FLASH (v1.2.11 [48]). Operational taxonomic units (OTUs) were clustered at 97% sequence similarity using UPARSE (v7.1; [49]), and chimeric sequences were removed during the clustering process. Taxonomic classification of representative sequences from each OTU was performed using the RDP Classifier (v2.2 [50]) against the SILVA 16S rRNA reference database, with a confidence threshold of 0.7.

### Data analysis

In this study, data in the results tables are presented as means ± standard error of the mean (SEM). Using the mixed model procedure for SAS 9.4 software (SAS Institute Inc., Cary, NC, USA), the impact of AMCB supplementation in an early-lactation diet on the DMI, milk yield, milk composition, blood metabolites, and rumen fermentation parameters of dairy cows was statistically investigated. When a significant interaction between treatment and time was observed, graphs were generated using GraphPad Prism 9. The following model was employed to assess the interaction between the treatment and control groups:


$$Y=\mu +T_{i}+G_{j}+TG_{ij}+E_{ijKl}$$

where *Y* represents the dependent variable, μ is the overall mean, *T*_*i*_ denotes the effect of time, *G*_*j*_ represents the group effect, *TG*_*ij*_ captures the interaction effect between *T* and *G*, and *E*_*ijKl*_ refers to the random residual error. Differences were considered statistically significant at *P* < 0.05, while a trend toward statistical significance was noted for 0.05 < *P* < 0.10.

The baseline data for the corresponding variables were included in the respective models as covariates. If the covariates did not exhibit a significant effect, they were excluded from the final model to optimize model structure.

Alpha diversity was assessed for each sample to evaluate microbial diversity within individual samples. Group differences in microbial α-diversity indices, as well as differences at the phylum and genus levels, were analyzed using the Kruskal-Wallis test, followed by Welch’s test for multiple comparisons, and plotted with origin 2024. Beta diversity analysis was conducted using the Bray-Curtis distance metric, and principal coordinates analysis (PCoA) was employed for visualization. Group differences in β-diversity were tested using PERMANOVA (Adonis) and annotated accordingly.

Correlation analysis and plotting of significantly altered rumen microbial taxa and phenotypic indicators were performed using https://www.chiplot.online/. Pearson’s correlation coefficients (r) were calculated, and results with *P* < 0.05 were considered statistically significant. Heat maps were used to visualize the strength and direction of correlations.

## RESULTS

### DMI and milk production

The impact of AMCB supplementation on the DMI and milk production of dairy cows during peak lactation is shown in Table 3.

**TABLE 3:** Effects of AMCB addition on dry matter intake and milk performance

Item	Group^*a*^		SEM	*P*-value		
	CON	EXP		Group	Time	Group × Time
DMI, kg/d	22.40	24.73	0.17	＜0.01	＜0.01	＜0.01
Milk yield, kg/d						
Actual	48.96	50.47	0.13	＜0.01	＜0.01	0.06
Fat-corrected	45.17	48.20	0.47	＜0.01	＜0.01	0.10
Energy-corrected	48.40	49.87	0.53	0.05	0.10	0.31
Efficiency, kg/kg						
Actual milk yield/DMI	2.03	2.02	0.02	0.95	0.07	0.12
FCM yield/DMI	1.71	1.74	0.21	0.52	0.12	0.29
ECM yield/DMI	1.96	2.12	0.04	0.04	＜0.01	＜0.01
Milk fat, %	3.65	3.73	0.04	0.16	0.16	0.37
Milk protein, %	3.22	3.45	0.01	＜0.01	＜0.01	＜0.01
Milk lactose, %	5.07	5.11	0.00	＜0.01	＜0.01	＜0.01
Total milk solids, %	12.37	12.99	0.34	0.23	＜0.01	0.21
Milk SCC, ×1,000 /mL	104.95	73.57	5.39	＜0.01	＜0.01	＜0.01
MUN, mg/dL	14.74	13.95	0.30	0.08	＜0.01	＜0.01
Milk fat yield, kg/d	1.73	1.88	0.03	＜0.01	＜0.01	0.04
Milk protein yield, kg/d	1.54	1.72	0.01	＜0.01	＜0.01	＜0.01
Milk lactose yield, kg/d	2.43	2.56	0.01	＜0.01	＜0.01	0.08

^*a*^ Group: CON, control; EXP, the group with AMCB-supplemented. Each group comprised 15 participants, with results reported as mean ± standard error of the mean (SEM).

**Fig 1:**
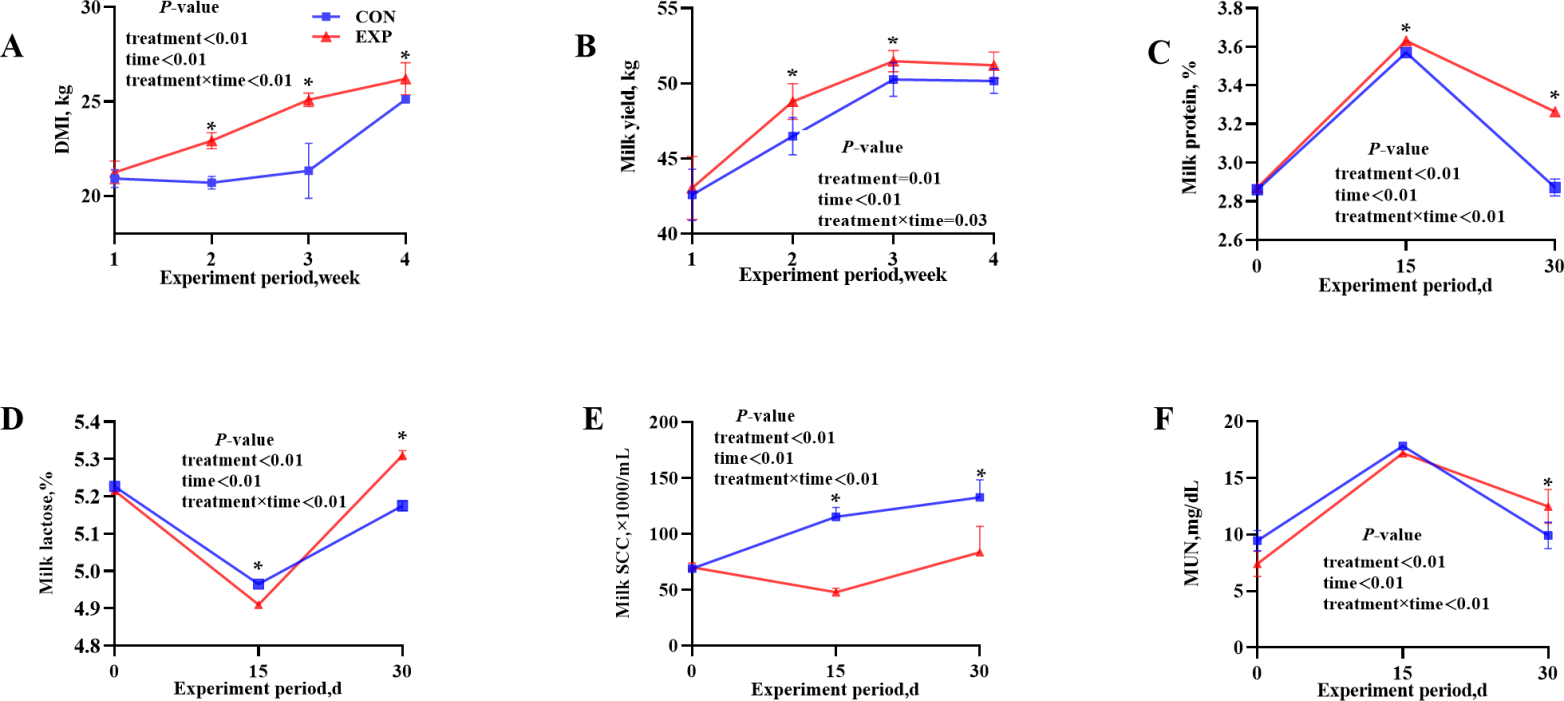
(**A–F**) Effects of AMCB treatment, time, and their interaction on dry matter intake, milk yield, and lactation performance. *P < 0.05, and values are presented as mean ± standard error of the mean (SEM).

Statistical analysis indicated that AMCB supplementation significantly increased DMI, milk yield, ECM, and FCM production compared with the control group (*P* < 0.05). Although no significant differences were observed in milk production/DMI and FCM/DMI between the two groups, ECM/DMI was significantly higher in the AMCB-supplemented group (*P* < 0.05), suggesting improved energy-adjusted milk production efficiency. Moreover, cows receiving AMCB exhibited reduced SCC and significant improvements in milk protein and lactose concentrations, as well as their respective yields and fat yield (*P* < 0.05), while it decreased milk MUN levels, albeit without statistical significance (0.05 < *P* < 0.10). Figure 1 illustrated a substantial interaction effect between treatment and time on daily milk protein synthesis, lactose concentration, SCC, MUN, DMI, and milk output.

### Serum variables

**Fig 2:**
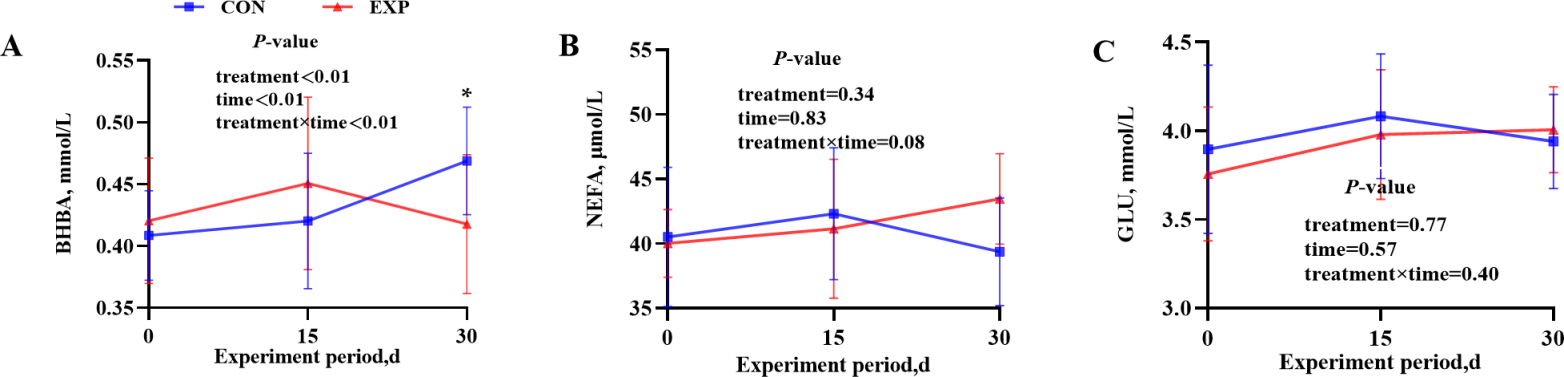
(**A–C**) Effects of AMCB treatment, time, and their interaction on serum BHBA, NEFA, and GLU concentrations. *P < 0.05, and values are presented as mean ± standard error of the mean (SEM).

The statistical results for serum variables are shown in Table 4. AMCB supplementation had no statistically significant effect on serum concentrations of energy and nitrogen metabolism-related parameters, including NEFA, TC, TG, Cr, TP, ALB, GLB, and BUN. According to the results of the two-way interaction between treatment and time, at d 30, BHBA concentration was significantly higher in the group without AMCB supplementation compared to the supplemented group (Fig. 2) (*P* < 0.05).

**TABLE 4:** Effects of AMCB addition on serum indexes

Item	Group^*a*^		SEM	*P*-value		
	CON	EXP		Group	Time	Group × Time
Metabolic variables						
BHBA, mmol/L	0.44	0.44	0.01	0.64	0.67	0.03
NEFA, umol/L	42.29	40.86	1.04	0.34	0.83	0.08
GLU, mmol/L	3.99	4.02	0.07	0.77	0.57	0.40
TC, mmol/L	5.54	5.43	0.24	0.73	0.87	0.52
TG, mmol/L	0.20	0.19	0.01	0.11	0.42	0.36
Cr, umol/L	78.56	81.37	1.18	0.11	＜0.01	0.14
TP, g/L	73.96	73.75	0.99	0.88	0.23	0.33
ALB, g/L	34.63	34.67	0.34	0.93	0.58	0.02
GLB, g/L	39.11	39.30	1.07	0.90	0.34	0.88
BUN, mmol/L	6.65	6.28	0.18	0.18	0.12	0.07
Antioxidant variables						
SOD, U/mL	45.59	45.04	0.85	0.65	0.32	0.23
MDA, nmol/mL	1.56	1.53	0.03	0.48	0.80	0.43
H_2_O_2_, mmol/L	23.82	23.86	1.19	0.98	0.46	0.25
CAT, U/ML	17.60	17.32	0.29	0.51	0.36	0.90
Immunity variables						
IgG, mg/mL	17.76	17.98	0.42	0.71	0.01	0.06
IgA, ug/mL	581.34	544.83	15.82	0.11	0.10	0.82
IL-6, ng/L	445.30	439.36	11.80	0.72	0.23	0.33
GM-CSF, pg/mL	47.29	47.04	0.98	0.86	0.12	0.87
Liver function indexes						
ALT, U/L	31.47	26.35	1.09	＜0.01	0.10	0.98
AST, U/L	94.61	77.17	5.10	0.02	0.49	0.91
ALP, U/L	40.64	46.29	3.80	0.30	0.84	0.25
T-BIL, umol/L	4.18	3.94	0.33	0.62	0.04	0.40

^*a*^ Group: CON, control; EXP, the group with AMCB-supplemented. Mean ± standard error of the mean (SEM) [n = 10 per experimental group] is how the results are displayed.

### Rumen fermentation

The statistical analysis of rumen fermentation parameters, presented in Table 5, indicated that the supplementation of AMCB had no statistically significant effect on ruminal pH, the concentration of individual and total volatile fatty acids (TVFA), and the acetate-to-propionate ratio (A:*P*). However, the addition of the alkaline composite buffer significantly elevated NH_3_-N concentrations (*P* < 0.05), while its impact on MCP yield was not statistically significant.

**TABLE 5:** Effects of AMCB addition on rumen fermentation parameters

Item	Group^*a*^		SEM	*P*-value		
	CON	EXP		Group	Time	Group × Time
pH	6.48	6.53	0.08	0.64	0.16	0.90
NH_3_-N, mg/dL	15.61	17.61	0.63	0.04	0.44	0.55
MCP, μg/mL	544.96	502.84	33.72	0.42	0.03	0.07
Total VFA, mmol/L	100.52	100.27	3.42	0.96	0.06	0.70
Individual VFA, mmol/L						
Acetic acid	59.65	62.00	3.17	0.61	0.03	0.62
Propionic acid	27.22	24.20	1.54	0.19	0.33	0.09
Butyric acid	10.33	11.08	0.87	0.55	0.32	0.79
Isobutyrate	0.47	0.48	0.01	0.20	0.49	0.92
Valerate	1.08	1.15	0.02	0.06	0.33	0.14
Isovalerate	1.49	1.58	0.04	0.12	0.91	0.44
Acetic acid/propionic acid	2.32	2.61	0.21	0.97	0.52	0.12

^*a*^ Group: CON, control; EXP, the group with AMCB-supplemented. Mean ± standard error of the mean (SEM) [n = 8 per experimental group] is how the results are displayed.

### Rumen microbial profiles

**Fig 3:**
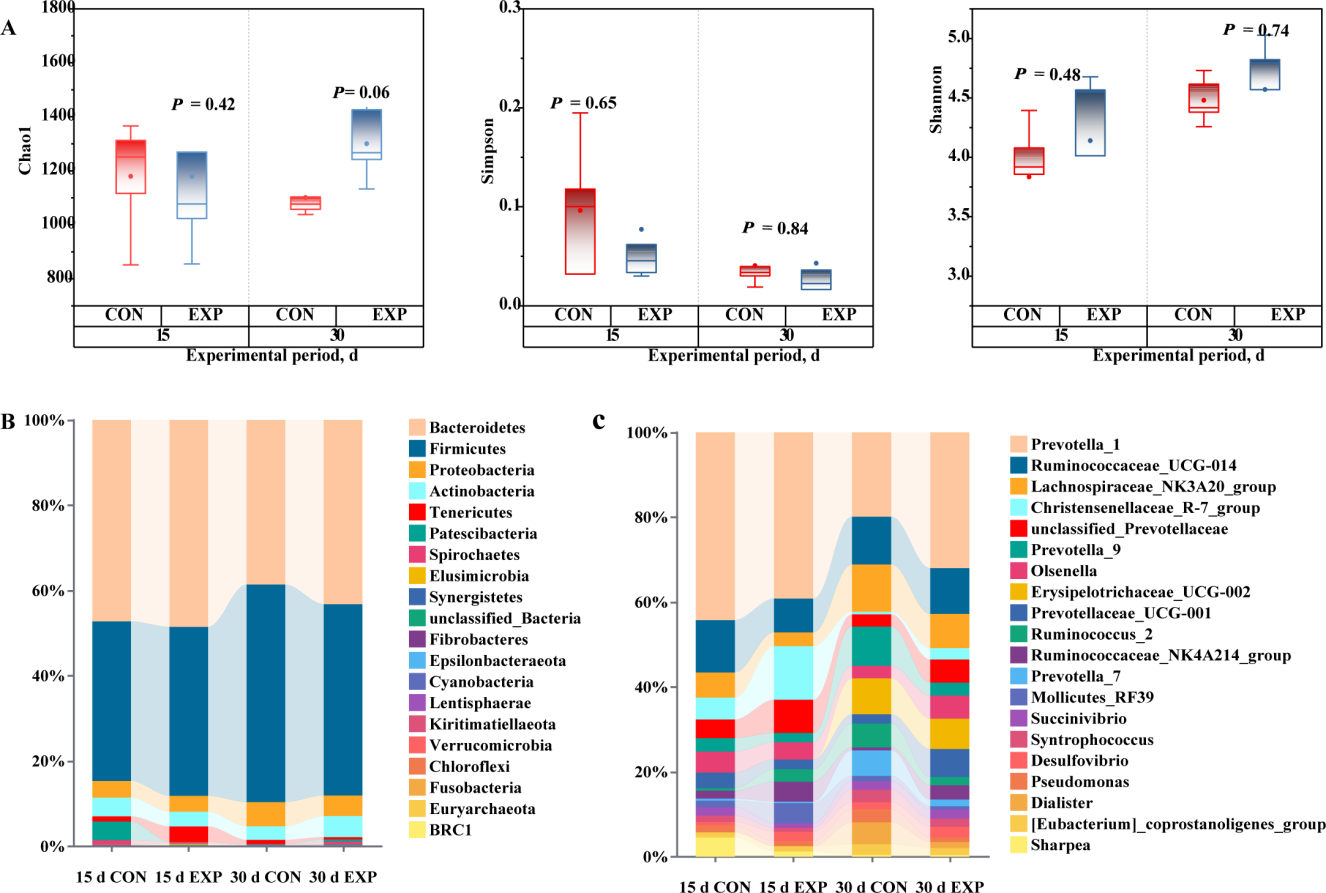
The influence of AMCB feeding on rumen microbial communities was investigated. (**A**) Alpha diversity of rumen bacterial communities on days 15 and 30 of the experiment. (**B**) Taxonomic distribution at the phylum level (top 20 phyla) and (**C**) at the genus level (top 20 genera), indicating the relative abundance of microbial species. *n* = 8 per experimental group.

Following the investigation into milk performance, blood metabolism, and rumen fermentation, this study continued to examine the impact of AMCB supplementation on the rumen microbiota. Rank-abundance curves at d 15 and 30 of the experiment stabilized and plateaued, illustrating that the sequencing depth was competent to capture the microbial communities within the samples. No significant influence was observed in the alpha diversity indices characterizing the rumen microbiota; however, by d 30 of the experiment, supplementation with AMCB led to an increase in the Chao1 index of the rumen microbiota, though the effect was not statistically evident (0.05 < *P* < 0.10) (Fig. 3A). At the phylum level, Firmicutes and Bacteroidetes dominated the rumen microbiota, with a shift in relative abundance throughout the study: Firmicutes increased while Bacteroidetes declined. Firmicutes, Bacteroidetes, Proteobacteria, and Actinobacteria (with a relative abundance of >1% in at least one group) were identified as core phyla (Fig. 3B). At the genus level, *Prevotella_*1 was the predominant genus, followed by *Ruminococcaceae_*UCG-014 (Fig. 3C).

**Fig 4:**
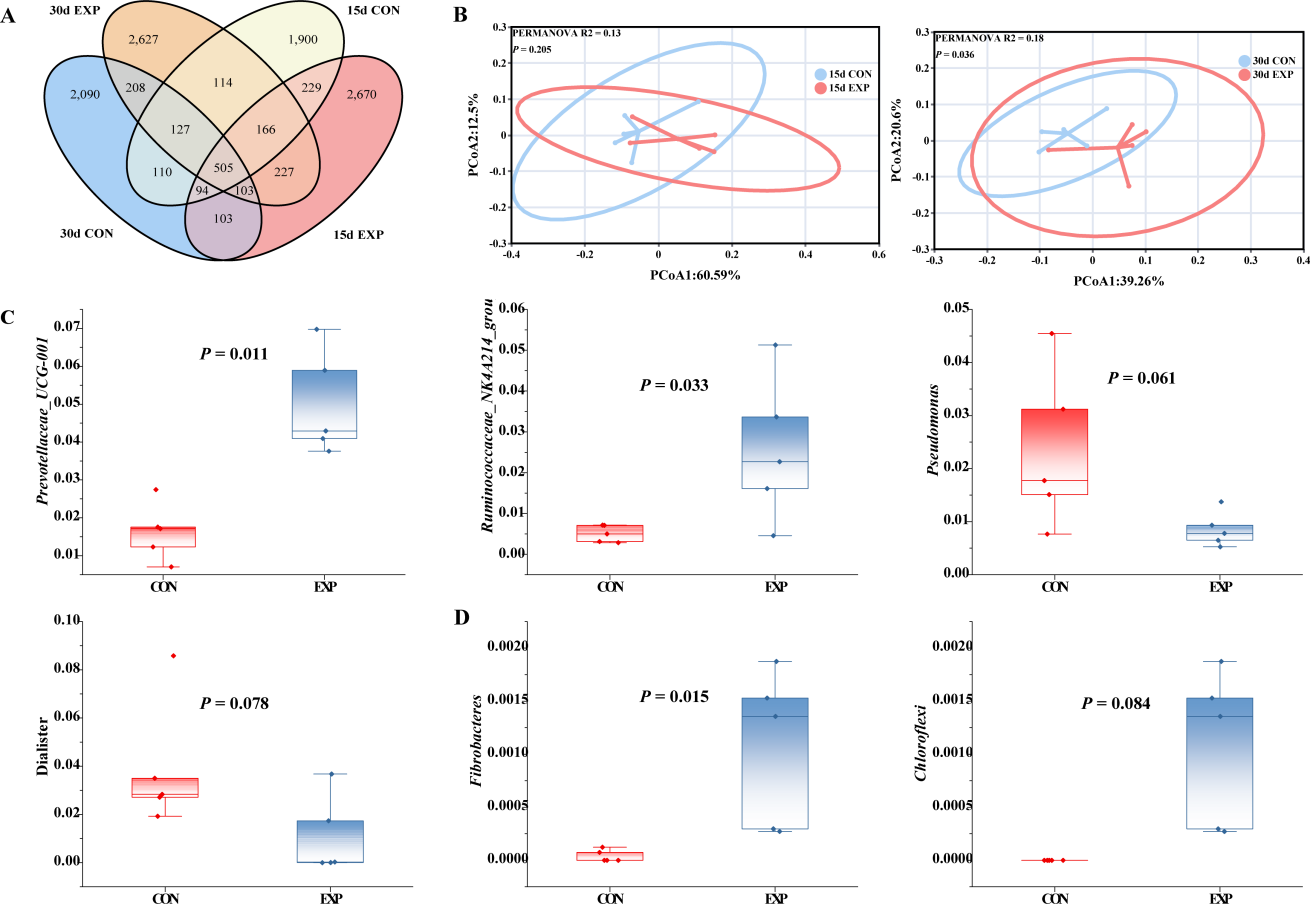
Impacts of AMCB upon the rumen microbial profile. (**A**) Venn diagram depicting the shared OTUs between groups on d 15 and 30 of the experiment. (**B**) PCoA clustering analysis illustrating β-diversity. (**C**) Genera showing significant differences or trends towards significance between the two groups at the genus level on d 30 (top 20 genera). (**D**) Phyla exhibiting significant differences or trends towards significance comparing the two groups at the phylum level on d 30 (top 20 phyla). *n* = 8 per experimental group.

On the d 15 and 30 of the experimental period, the AMCB-supplemented group shared a total of 1,001 ASVs, while the control group shared 836 ASVs (Fig. 4A). The overlapping ellipses in the PCoA plot indicated that, at d 15, the microbial profiles of the two groups were not sufficiently distinct to form separate clusters. However, by d 30, the treatment and control groups occupied different regions in the ordination space, with most of the variation captured along PCoA1. PERMANOVA analysis confirmed a substantial variation in microbial community composition between the two groups (R² =0.18, *P* = 0.027) (Fig. 4B).

At d 15, AMCB supplementation did not induce any significant changes in bacterial phyla or genera in the rumen. However, after an additional 15 days of feeding, AMCB significantly increased the relative abundance of Fibrobacteres (*P* < 0.05), while the abundance of Chloroflexi declined (tendency, *P* = 0.08) (Fig. 4D). At the genus level, AMCB supplementation significantly elevated the relative abundance of *Prevotellaceae*_UCG*-*001 and *Ruminococcaceae_*NK4A214_ group, while *Pseudomonas* and *Dialister* indicated a downward trend in abundance (0.05 < *P* < 0.10) (Fig. 4C).

**Fig 5:**
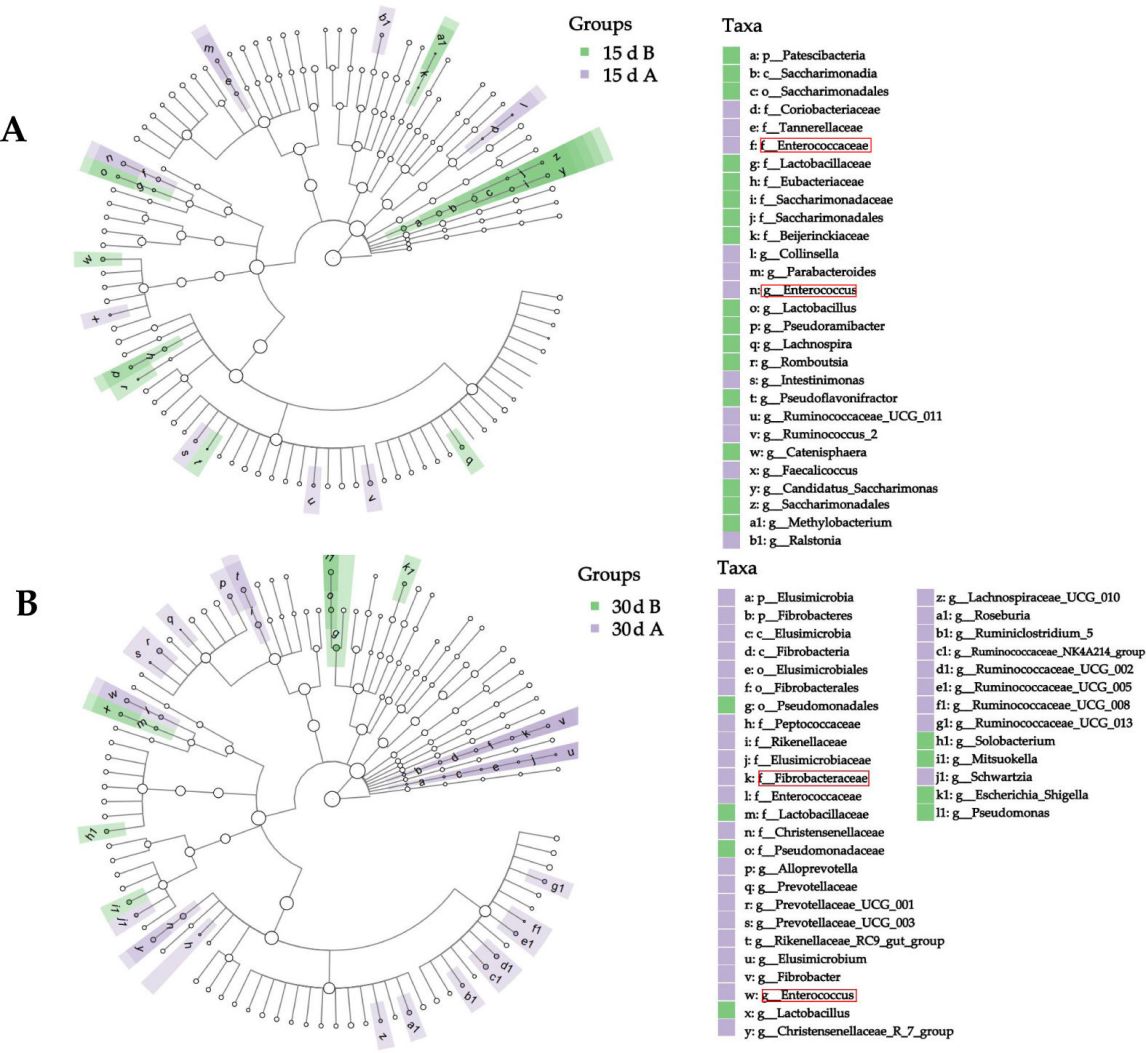
LEfSe analysis at 15 d (**A**) and 30 d (**B**) of the experiment showing differential microbial taxa.

LEfSe analysis identified 29 and 39 distinct microbial taxa at 15 and 30 d, respectively. On d 15, the AMCB-supplemented group exhibited significant enrichment primarily within the phylum Firmicutes and the family Ruminococcaceae, while the control group showed enrichment mainly within the phylum Patescibacteria and the family Saccharimonadaceae (Fig. 5A). By d 30, AMCB supplementation facilitated the enrichment of ruminal bacteria within the phylum Bacteroidetes and the family Prevotellaceae, as well as within the phylum Firmicutes and the family Ruminococcaceae, whereas the control group was predominantly enriched in the phylum Proteobacteria and the family Pseudomonadaceae (Fig. 5A). A closer examination of Fig. 4 revealed that AMCB supplementation consistently enriched ruminal bacteria in the family Enterococcaceae and the genus *Enterococcus* at both time points.

**Fig 6:**
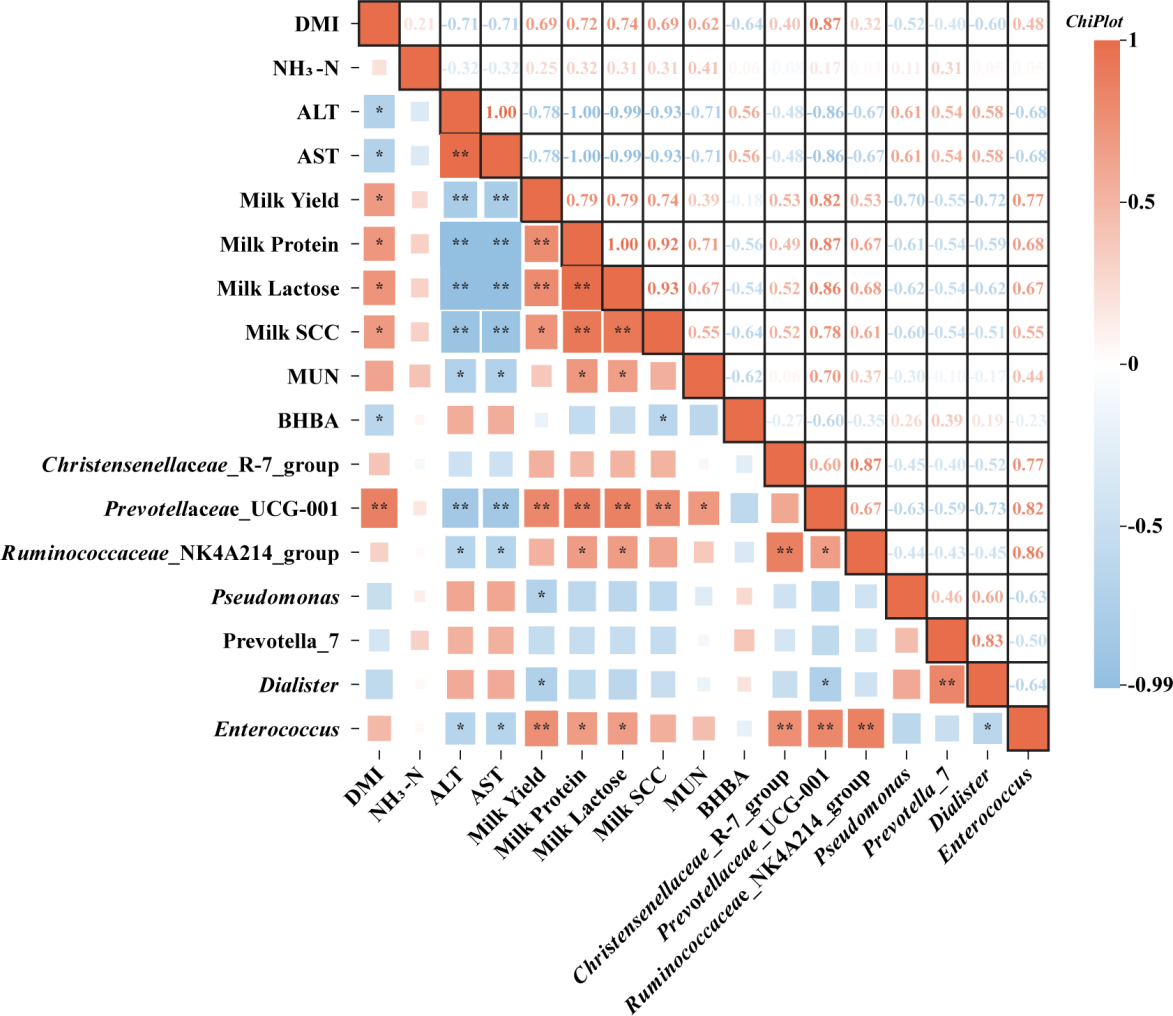
Correlation analysis between significantly abundant rumen microorganisms (top 20 genera) and markedly altered phenotypic indicators at d 30. DMI, dry matter intake; NH_3_-N, ammonia nitrogen; ALT, alanine aminotransferase; AST, aspartate aminotransferase; SCC, somatic cell count; MUN, milk urea nitrogen; BHBA, β-hydroxybutyric acid.

Correlation analysis between rumen microorganisms, which showed significant or trending differences in relative abundance between the two groups, and the phenotypic indicators that exhibited significant differences at d 30 (r > 0.6, *P* < 0.05) revealed strong correlations. Specifically, *Prevotellaceae*_UCG-001, *Ruminococcaceae*_NK4A214_group, and *Enterococcus* were significantly negatively correlated with serum ALT and AST levels while displaying significant positive correlations with milk protein content and lactose concentration (r ≥ 0.67, *P* < 0.05). *Prevotellaceae*_UCG-001 also exhibited a positive correlation with DMI, milk yield, MUN, and SCC (r ≥ 0.7, *P* < 0.05) (Fig. 6).

## DISCUSSION

It has been shown that DMI in dairy cows exhibits a quadratic response to the supplementation of alkaline buffers such as zeolite. DMI remained stable at moderate supplementation levels (below 300 g/d/cow); however, this pattern reversed once the dosage hit 400 g/d/cow, leading to a sharp drop in consumption (51). Notably, the AMCB feed additive in the current study considerably raised DMI during the peak lactation phase, aligning with the conclusions of Hu and Murphy (52) and Kawas et al. (53), which demonstrated that the inclusion of bicarbonates in diets where corn silage is a major component positively influenced DMI. According to the descriptions by Russell and Chow (54), bicarbonate supplementation increased rumen osmolarity, resulting in greater water intake and accelerated rumen outflow. Consequently, the faster evacuation of ruminal contents stimulated greater feed intake in dairy cows.

As shown in earlier research, the increase in DMI provides the udder with an adequate energy supply, thereby supporting higher milk production. Therefore, there is a strong link between DMI and milk output (55). Our experiment corroborated this relationship, as the addition of AMCB resulted in substantial increases in both milk yield and ECM. Notably, the integration of silicates or bicarbonates into dairy cow diets has been reported to enhance average milk production (56, 57), particularly when corn silage was a major dietary component (58). The positive impact of buffer supplementation on milk yield was especially pronounced under these feeding conditions. The relationship between BHBA and energy balance in early lactation has been well established, reflecting the mobilization of body reserves and the allocation of nutrients for milk production (59). By d 30, dairy cows in the CON group displayed significantly elevated serum BHBA concentrations compared to those receiving AMCB supplementation, a finding consistent with the observation that, despite markedly lower DMI, milk yield in the non-supplemented group remained stable at d 30, indicating a compensatory reliance on body fat mobilization to meet energy demands for milk production. Such mobilization likely intensified ketogenesis, thereby contributing to the elevated BHBA levels observed in the CON group (60). In the present study, AMCB did not benefit carbohydrate fermentation in dairy cows, nor did it alter the fermentation pattern. Consequently, there were no discernible variations in the levels of acetate or milk fat, which aligns with findings from prior research (61, 62). Similarly, Tucker et al. (63) reported that buffers did not affect milk fat content during early to mid-lactation, while a general increase was observed during late lactation. Moreover, the AMCB group exhibited a significant increase in milk fat yield, attributed to the enhanced milk production observed in this group. In our investigation, adding AMCB substantially cut down the amount of SCC in milk. Numerous authors have documented a strong negative correlation between SCC and daily milk yield (64, 65), which is closely linked to the damage inflicted on mammary tissue by exogenous pathogenic bacteria and bacterial endotoxins.

The concentration of NH_3_-N in the rumen is influenced by the degradable protein content of the substrate and the activity of rumen microorganisms (66). The rumen’s NH_3_-N levels rose substantially in the present investigation when AMCB was applied, which was partially explained by the elevated abundance of the genus *Prevotellaceae*_UCG-001, thereby facilitating protein degradation. However, no statistically significant change was observed in the yield of MCP (67), potentially due to limitations in fermentable energy. Previous studies in lactating goats have demonstrated that dietary alkaline buffers can modulate hepatic nitrogen metabolism by promoting amino acid synthesis while inhibiting urea production (68). Specifically, buffer supplementation increased hepatic amino acid synthesis, as reflected by higher free amino acid concentrations in hepatic venous blood relative to systemic circulation. These findings suggest that the elevated ruminal NH₃-N induced by buffer supplementation may have been redirected from urea production toward amino acid utilization and protein synthesis. Consistently, serum ALT and AST—markers of liver function and metabolic stress (69)—were significantly reduced following AMCB supplementation in our study, indicating alleviated hepatic metabolic load and potentially improved nitrogen utilization. Consistent with these hepatic metabolic changes, milk protein content was also significantly increased in the AMCB group, further suggesting enhanced amino acid availability for protein synthesis at the mammary level. This enhancement in milk protein synthesis likely underpinned the improved ECM production efficiency. While milk production/DMI and FCM/DMI remained unchanged, ECM/DMI was significantly higher in the AMCB group, largely due to the increased milk protein yield, which contributed to greater energy-corrected milk output per unit of intake.

Milk protein is primarily synthesized by the mammary glands of lactating cows utilizing circulating plasma amino acids (70), with epithelial cells of the mammary gland employing transporters to facilitate the uptake of amino acids (71, 72). He et al. (73) demonstrated that the addition of buffer facilitated the transport of amino acids, allowing for greater availability of amino acids for milk protein synthesis. This finding elucidated the significant improvement in milk protein content observed in our experiment, aligning with the conclusion summarized by Hu and Murph (74). In addition, previous studies have reported that the use of potassium-rich multi-element buffers resulted in higher milk protein content (75, 76), which aligned with our current findings using AMCB. Given that various minerals influence osmotic balance (77), it can be hypothesized that the effects of potassium-containing AMCB are attributable to the interactions among the constituent minerals. However, more research is warranted to clarify the specific mechanisms behind these interactions. Typically, the concentration of MUN is inversely correlated with milk protein content while exhibiting a positive correlation with BUN level (78). In this study, AMCB led to a reduction in the urea nitrogen content in both milk and serum, albeit without statistical significance. Nonetheless, these results indicated that the supplementation of alkaline mineral buffer may have enhanced protein utilization to some extent, reflecting a favorable state of nitrogen metabolism within dairy cows (79). The addition of buffer to diets is typically intended to mitigate sudden decreases in ruminal pH; however, in this experiment, the control group already maintained a normal ruminal pH of approximately 6.5. Other studies have similarly demonstrated that the incorporation of silicates did not significantly impact ruminal pH, and many studies have not revealed any increase in ruminal pH following the supplementation of sodium bicarbonate.

The gastrointestinal microbiota constitutes a complex communimicrobial interactions within the rumen that plays a crucial role in health, metabolism, and immune function. Supplementation with AMCB has shown a tendency to increase rumen microbial species abundance. Bacteroidetes and Firmicutes emerged as the dominant groups at the phylum level, collectively accounting for an average of 87.65% of the 16S rRNA gene sequences. Previous studies have also identified these phyla as predominant in rumen bacteria (80, 81). Bacteroidetes primarily mediate the degradation of non-fibrous substances, and they are actively involved in the metabolism of amino acids and fatty acids, which is crucial for improving the efficiency of nitrogen consumption (82). Firmicutes are recognized for their fiber-degrading capabilities, which allow them to break down complex plant cell walls (83). The Firmicutes-to-Bacteroidetes (F/B) ratio plays a pivotal role in maintaining gastrointestinal homeostasis. In the present study, supplementation with the alkaline composite buffer resulted in a 27.38% reduction in the F/B ratio by the end of the experiment, accompanied by a 10.67% increase in the relative abundance of Bacteroidetes. This microbial shift may reflect a transition toward metabolic pathways that prioritize nitrogen assimilation over fiber fermentation. Such a change could account for the observed improvements in milk protein concentration and nitrogen utilization efficiency, even in the absence of significant differences in VFA production.

At the genus level, *Prevotella-*1, *Ruminococcaceae*_UCG-014, *Lachnospiraceae_*NK3A20*_*group, and *Christensenellaceae*_R-7_group were identified as common dominant bacteria in this study, consistent with previous research findings (35, 84). Among the genera exhibiting significant differences, *Prevotellaceae*_UCG*-*001 had the highest relative abundance, averaging 3.32% of the OTUs in the samples. *Prevotella* is a well-established genus closely linked to bile acid secretion and the metabolism of protein digestion and absorption (85). The observed rise in the relative abundance of *Prevotellaceae*_UCG-001 corresponds with the higher NH_3_-N concentrations in the rumen of cows provided AMCB in the present study, indicating an enhanced degradation of amino acids and intensified deamination processes (86). This finding aligned with prior research investigating the effects of magnesium hydroxide on the composition of the rumen microbiota in dairy cows (87). Correlation analysis showed strong positive associations between *Prevotellaceae_UCG-001* and NH₃-N, MUN (r = 0.70 and 0.67), milk yield, and protein content, suggesting that despite increased proteolytic nitrogen release, much of the liberated nitrogen was efficiently recycled into anabolic pathways supporting milk synthesis, reflecting improved nitrogen utilization. This supports a mechanistic link whereby AMCB modulates rumen microbiota to improve nitrogen supply for milk synthesis.

*Ruminococcaceae*_NK4A214_group was one of only two genera among the top 20 ranked by relative abundance that exhibited significant differences in this study. It is well known that the *Ruminococcaceae* family can effectively break down complex plant components, particularly cellulose, thereby promoting the production of VFAs (88). However, our study did not detect any statistically significant changes in VFA concentrations, indicating that the observed variations in *Ruminococcaceae*_NK4A214_group were insufficient to induce any differences related to ruminal fermentation. Nonetheless, consistent with Zhang et al. (43), we found a significant positive correlation between this genus and milk protein content (r = 0.67, *P* < 0.05), supporting its association with milk protein yields. At both 15 and 30 days of the experiment, *Enterococcus* was identified as a unique biomarker in the treatment group through LEfSe analysis. As a member of the lactic acid bacteria, *Enterococcus* encompasses approximately 50 known species, with *Enterococcus faecalis* and *Enterococcus faecium* being the most commonly associated species with the gastrointestinal tract of dairy cows and frequently isolated from dairy products (8932). A study feeding isolated *Enterococcus faecium* to lactating cows found that it significantly improved DMI and milk production while also showing the potential to enhance liver function and overall health in the cows (90). According to these results, *Enterococcus* may be essential for optimizing the performance of dairy cows, warranting further exploration into its mechanisms of action and potential applications in dairy nutrition. Furthermore, it is important to recognize that microbial interactions within the rumen create a complex ecosystem. Dietary interventions present a novel strategy for manipulating the rumen microbiota, thereby regulating the health and output of dairy cows during peak lactation.

### Conclusion

The incorporation of AMCB led to an improvement in DMI, which positively correlated with enhanced milk production. Furthermore, AMCB contributed to superior milk composition, resulting in elevated levels of both milk protein and lactose. Although the concentration of rumen NH_3_-N increased levels of BUN and MUN decreased, indicating that the increased abundance of certain protein-degrading bacteria optimized nitrogen utilization efficiency. In addition, supplementation with AMCB was associated with a reduction in serum concentrations of AST and ALT, suggesting a decreased hepatic burden. Collectively, these findings underscore the multifaceted advantages of AMCB in enhancing both the health and productivity of dairy cows.

## Data Availability

The raw sequencing data have been deposited in the NCBI Sequence Read Archive under BioProject accession number PRJNA1308777 (SRX30176470–SRX30176489).
